# A Comprehensive Data Mining-Based Study for Review of Animal Studies on Traditional Chinese Medicine Prescriptions for Treating Ulcerative Colitis

**DOI:** 10.2174/0118715303406481251029074257

**Published:** 2026-01-12

**Authors:** Juan Wang, Jia-Hui Xu, Meng Meng Zhang, Yao Li, Hui Min Xiao, Min Li

**Affiliations:** 1 The College of Pharmacy, Shaanxi University of Chinese Medicine, Xianyang 712046, China;; 2 College of Chemistry and Pharmacy, Northwest A&F University, Yangling, China

**Keywords:** Traditional Chinese medicine, classic prescriptions, ulcerative colitis, data mining, immune balance, immune disorders

## Abstract

**Background:**

Ulcerative colitis (UC) is a complex disease associated with immune dysregulation. Classic prescriptions (CP) of traditional Chinese medicine have a strong theoretical foundation and a long history of clinical use in treating UC. This study aims to uncover the intrinsic patterns and characteristics of these CPs.

**Methods:**

Data mining of CP literature for UC from VIP, CNKI, WanFang, and PubMed (2013–2023), which focused on herb usage frequency, properties, flavors, meridian tropisms, core herb pairs, and UC categories. Finally, the top ten CPs were further analyzed for clinical application.

**Results:**

A total of 62 prescriptions from 383 articles were analyzed, and the most frequently used prescriptions were Sishen pill, Huangqin decoction, and Shaoyao decoction. The greatest frequency herbs were *Glycyrrhizae Radix et Rhizoma, Coptidis Rhizoma, Paeoniae Radix Alba, and Atractylodis Macrocephalae Rhizoma*. Herbs were pungent, bitter, and sweet in flavor with properties mainly being warm, cold, and neutral and targeting the liver, spleen, stomach, and heart meridians. Core herb pairs included *Bupleuri-Poria, Atractylodis-Saposhnikoviae, Zingiberis-Jujubae, Phellodendri Chinensis Cortex -Coptidis Rhizoma, Pinelliae Rhizoma-Ginseng Radix et Rhizoma.* Through cluster analysis, the herbs were categorized into four distinct clusters. Furthermore, the CP primarily exerted therapeutic effects by alleviating inflammation, restoring mucosal barriers, and maintaining immune balance.

**Conclusion:**

Data mining identified Sishen Pill, Huangqin Decoction, and Shaoyao Decoction as the top three CPs for UC. The main mechanism of treating UC is by reducing inflammation, maintaining immune balance, and repairing the mucosal barrier.

## 1 INTRODUCTION

Ulcerative colitis (UC) is a non-specific disease with a complex etiology, including immune disorders, inflammation, and environmental factors [1-3]. Although the mortality rate of UC is not higher than that of the general population, symptoms such as diarrhea and rectal bleeding cause many patients to suffer from depression, anxiety, and sleep disorders, resulting in a lower quality of life and increased likelihood of being unable to work [4-6]. Moreover, UC patients with long-term immune dysregulation and chronic inflammatory stimulation have a higher risk of developing colorectal cancer. The risk of colon cancer by the age of 20 in UC patients is 4.5%, which is 1.7 times higher than that of the general population [7, 8]. Although Western countries are experiencing a plateau in UC incidence, its prevalence remains over 0.5%, much higher than in developing countries [9, 10]. Furthermore, the prevalence of UC in developing countries, including China and regions of Africa, continues to rise, posing enormous challenges to healthcare systems [11, 12].

In Chinese medicine, UC is classified under “diarrhea” and “dysentery,” and the use of classic prescriptions (CPs) for its treatment can be traced back to the Han Dynasty. As early as 1,800 years ago, the Wumei pill and Baitouweng decoction were recorded in the Treatise on Febrile Diseases for the treatment of UC. From the Yuan Dynasty, Zhu Zhenheng’s Danxi’s Experiential Therapy describes Baizhu Shaoyao powder, noting that “it treats conditions of liver exuberance and spleen deficiency characterized by intestinal rumbling, abdominal pain, and diarrhea” [13, 14]. Traditional Chinese Medicine (TCM) has demonstrated significant advantages in managing refractory diseases due to its minimal side effects, relatively low cost, and high patient compliance. In the 2023 edition of the International Clinical Practice Guidelines for the Treatment of UC with TCM, Shaoyao decoction, Baitouweng decoction, and Shenling Baizhu powder were recommended for treating UC with different syndromes by clearing heat and dampness, cooling blood and detoxifying, and invigorating spleen and qi [15].

CP has been widely validated for its safety and efficacy through long-term use and clinical practice. Several controlled experimental studies on the treatment of UC with classical TCM formulas have shown that their use, either alone or in combination with Western medicines, alleviates clinical symptoms and reduces the recurrence rate of UC [14, 16, 17]. Modern research indicates that CP inhibits UC progression by modulating immune responses, regulating intestinal flora, and repairing the mucosal barrier. For example, Huangqin decoction significantly improved general symptoms in dextran sulfate sodium (DSS)-induced UC in mice, with mechanisms potentially involving the regulation of immune response and intestinal flora imbalance, upregulation of amino acid metabolism, and activation of the mTOR pathway [18]. Gegen Qinlian decoction activated the AhR/IL-22 pathway to strengthen the intestinal mucosal immune barrier in UC mice [19]. Baitouweng decoction effectively mitigated oxazolone-induced inflammatory progression in murine models [20].

Although there is an abundance of literature on CP for the treatment of UC, the regularity and core pair medicines of CP remain to be fully elucidated. The emergence of data mining techniques provides a foundation for illustrating the medication patterns of classical TCM formulas [21]. Animal experiments offer a scientific basis for CP derived from clinical experience and can be combined with pharmacology, pathology, and molecular biology to elucidate the mechanisms of prescriptions used to treat UC [22]. In this study, data mining techniques were employed to analyze experimental literature on CP for UC from 2013 to 2023, aiming to explore the frequency, patterns, compatibility, and biological processes of CP. The R language was used for data mining to screen core pair medicines and generate new formulas through clustering. Furthermore, clinical cases of representative classical TCM formulas for UC treatment were analyzed. These data mining results provide a foundation for further research on CP and represent an important step before conducting more complex and costly clinical trials.

## 2 MATERIALS AND METHODS

### 2.1 Database Introduction

Data were systematically retrieved from four authoritative databases: China National Knowledge Infrastructure (CNKI, comprehensive coverage of Chinese academic journals), VIP Information Resources (VIP, specialized focus on traditional Chinese medicine and scientific literature), Wanfang Data Knowledge Service Platform (Wanfang, integration of patent achievements and clinical trial data), and PubMed (globally recognized biomedical database). Multiple search methods complement each other to avoid bias Fig. (**1**).

### 2.2 Data Source of the Clinical Section

The search period was set from January 2005 to May 2024 with disease keywords (“ulcerative colitis”, “colitis”) and medicine keywords (“Si Shen pill”, “Shenlingbaizhu san”, “Baitouweng decoction”, “Huangqin decoction”, “Shaoyao decoction”, “Ge Gen Qin Lian decoction”, “Wumei pill”, “Tongxieyao fang”, “Banxiaxiexin decoction”, “Yulian pill”) in the CNKI.

### 2.3 Data source of the Experiment Section

The search language was limited to English or Chinese, and the search period was set to January 2013-December 2023 with disease-related terms (“ulcerative colitis”, “colitis”) and drug-related terms (“Chinese medicine”, “pill”, “san”, “formula”, “decoction”, “dosage”) in the three Chinese databases (CNKI, VIP, Wanfang). Then, use (((((ulcerative colitis) OR (inflammatory bowel disease)) OR (UC)) OR (IBD)) OR (colitis)) AND ((((((formula) OR (prescription)) OR (Chinese medicine)) OR (herb)) OR (capsule)) OR (decoction)) to search on pubmed.

### 2.4 Inclusion Criteria

The clinical literature inclusion criteria were: 1) Journal literature published between January 2005 and May 2024; 2) Clinical type literature; 3) Study disease is UC with a specific disease model.

The experimental research literature inclusion criteria were: 1) Journal literature published between January 2013 and December 2023; 2) Experimental animal literature; 3) The study disease was UC, and there was a specific disease model; 4) Gavage administration.

### 2.5 Exclusion Criteria

The same exclusion criteria were applied for both experimental and clinical literature: (1) duplicate publications; (2) studies on prescription combinations or ethnomedicine; (3) literature for which the full text was unavailable; (4) review articles, studies on complications, theses (master’s or doctoral), and conference papers; and (5) studies focusing on the treatment of UC-related complications or comorbidities.

### 2.6 Database Establishment

The literature was searched according to the inclusion and exclusion criteria, and information on the composition of CP and related animal models was extracted. Data were entered into a database using Excel 2010. The names of Chinese medicines were standardized with reference to the Pharmacopoeia of the People’s Republic of China (2020 Edition). Herbs not included in the pharmacopoeia were standardized according to the Dictionary of Chinese Medicines and Zhonghua Bencao; for example, orange peel was standardized as Citri Reticulatae Pericarpium. Herbs with different preparation methods but derived from the same source and exhibiting similar therapeutic effects were classified under a single category; for instance, roasted Glycyrrhiza glabra was standardized as Glycyrrhiza glabra, and calcined Os Draconis was standardized as Os Draconis.

### 2.7 Data Analysis

Excel 2010 was used to count the basic information of UC, such as the frequency of medication patterns, four natures, five flavors, and meridian tropisms, and R-Studio was used to process the data for association rules, cluster analysis, and correlation analysis.

- The clinical research of several classical prescriptions in randomized controlled trials was analyzed, and the effective rate and syndrome types of UC of classical prescriptions were analyzed.
- The types and frequency of each TCM were counted, and the properties, flavors, and meridians were analyzed.
- Hierarchical clustering using the Ward function was performed to classify the top 23 Chinese medicinal herbs, and the results were visualized.
- Association analysis: association rules based on the Apriori algorithm were generated by three indicators: confidence, support, and promotion.
- Phi analysis: The phi algorithm was used to analyze the top 23 TCM to explore the degree of correlation between the two TCM.

## 3 FORMULARY ANALYSIS

**Fig. (2):**
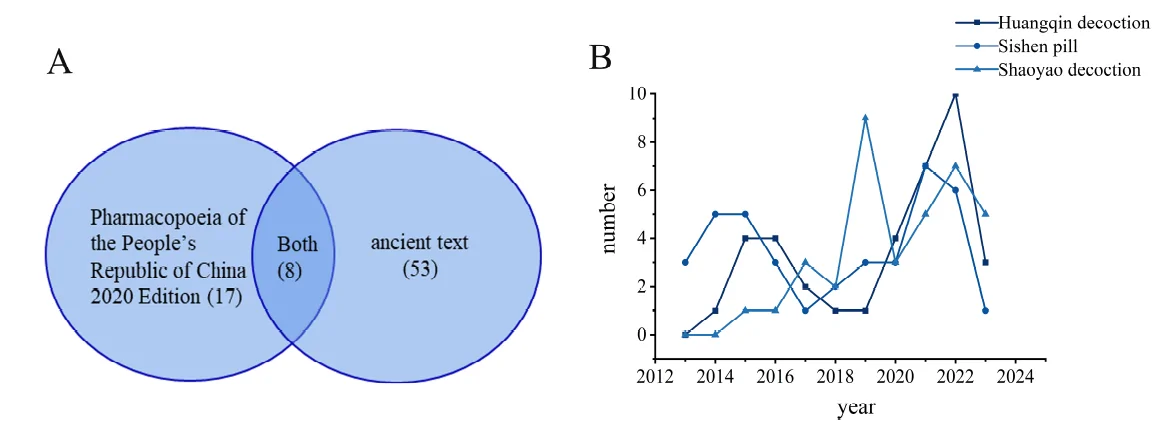
The prescription information for the treatment of UC. (**A**) Graph of the prescription derived; (**B**) The number of publications of the top three prescriptions.

Following the mentioned exclusion criteria, a total of 383 relevant studies on the treatment of UC with classical TCM formulas in animal models were identified, which encompassed 62 prescriptions. Notably, 17 of these prescriptions are recorded in the “Pharmacopoeia of the People’s Republic of China 2020 Edition”, and 53 have been sourced from ancient texts (Fig. **2A**). The most frequent were derived from “Treatise on Febrile Diseases”, accounting for 13 formulas. The top three classical TCM formulas are Sishen pill, Huangqin decoction, and Shaoyao decoction, each with substantial research over 30 studies (Table **1**), and the analysis of the research years indicates a notable upswing in studies on Shaoyao decoction in 2019, and the peak of investigative activity for Huangqin decoction and Sishen pill was observed in the years 2021 and 2022 (Fig. **2B**).

## 3.1 Frequency Analysis of Herbs

The cumulative frequency of the use of Chinese medicines was 383 times, involving 128 herbs. The greatest frequency of use in the prescription was *Glycyrrhizae Radix et Rhizoma*, which appeared 29 times (46.77%), and there were 9 herbs with ≥10 times of use, including *Glycyrrhizae Radix et Rhizoma, Coptidis Rhizoma, Paeoniae Radix Alba, Atractylodis Macrocephalae Rhizoma, Ginseng Radix et Rhizoma, Zingiberis Rhizoma, Jujubae Fructus, Angelicae Sinensis Radix, Scutellariae Radix*. A total of 23 herbs were used more than 5 times and were analyzed as high-frequency herbs in Table **2**.

## 3.2 Analysis of Drug Properties

**Fig. (3):**
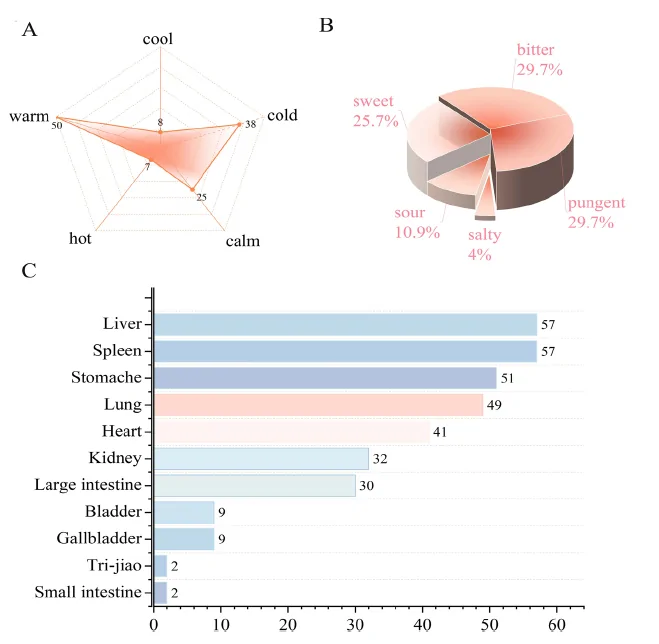
The drug properties in the prescriptions for the treatment of UC. (**A**) The four properties; (**B**) The five flavors: (**C**) The meridian tropism.

In terms of the herbs’ four properties, most were classified as warm, cold, or neutral, with occurrence frequencies of 50, 38, and 25, respectively (Fig. **3A**). Among the five flavors, pungent and bitter herbs predominated, each accounting for 29.7%, while sweet-flavored herbs comprised 25.7%. In contrast, salty and sour herbs were less frequently used (Fig. **3B**). Regarding meridian tropism, herbs targeting the liver, spleen, and stomach channels were particularly common, suggesting that the pathogenesis of UC may be closely associated with these organs (Fig. **3C**).

## 3.3 Association Rules

The Apriori algorithm was used to analyze the association rules of 128 herbs. The support degree was ≥ 0.08—indicating that the probability of the co-occurrence of any two items is at least 8%. The confidence degree was ≥ 0.7—signifying that the minimum probability of the subsequent item appearing given the antecedent is 70%. The lift > 1 indicates that the association rule was meaningful if the lift degree was >1. Using the function to obtain the top 10 association rules in Table **3**, *Glycyrrhizae Radix et Rhizoma* and *Ginseng Radix et Rhizoma* appear and are often used together with other herbs. *Glycyrrhizae Radix et Rhizoma* is predominantly utilized for its harmonizing properties in TCM formulations, and the herbal pair with *Glycyrrhizae Radix et Rhizoma* was excluded in this study. It is found that the combination of {*Pinelliae Rhizoma*} => {*Ginseng Radix et Rhizoma*}, {*Saposhnikoviae Radix}* => {*Atractylodis Macrocephalae Rhizoma*}, and {*Phellodendri Chinensis Cortex*} => {*Coptidis Rhizoma*} was also used for the treatment of UC. The visualized association rules are shown in Fig. (**4**), the plot function where the size of the graphic is related to the confidence level, and the shade of color represents the strength of the lift value.

## 3.4 Cluster Analysis

**Fig. (5):**
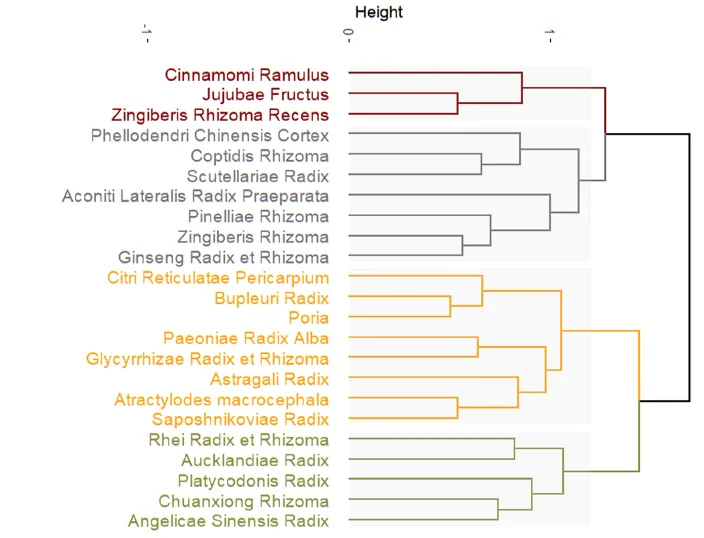
Cluster analysis tree diagram.

High-frequency herbs used ≥5 times were subjected to cluster analysis, resulting in four distinct clusters (Fig. **5**). The first cluster includes *Cinnamomi Ramulus, Zingiberis Rhizoma Recens*, and *Jujubae Fructus*, primarily used to treat UC by warming yang and tonifying the body. The second cluster consists of heat-clearing and detoxifying herbs, such as *Coptidis Rhizoma, Scutellariae Radix*, and *Phellodendri Chinensis Cortex*, often combined with a modified *Renshen Fuzi* decoction. The third cluster involves formulas like Tong Xie Yao Fang combined with Yu Ping Feng Powder. The fourth cluster includes herbs such as *Aucklandiae Radix, Chuanxiong Rhizoma*, and *Angelicae Sinensis Radix*, which activate blood circulation and relieve pain. Additionally, the combination of Rhei *Radix et Rhizoma* and *Platycodonis Radix* treats UC by both clearing heat and dispelling damp-heat.

## 3.5 Phi Analysis

**Fig. (6):**
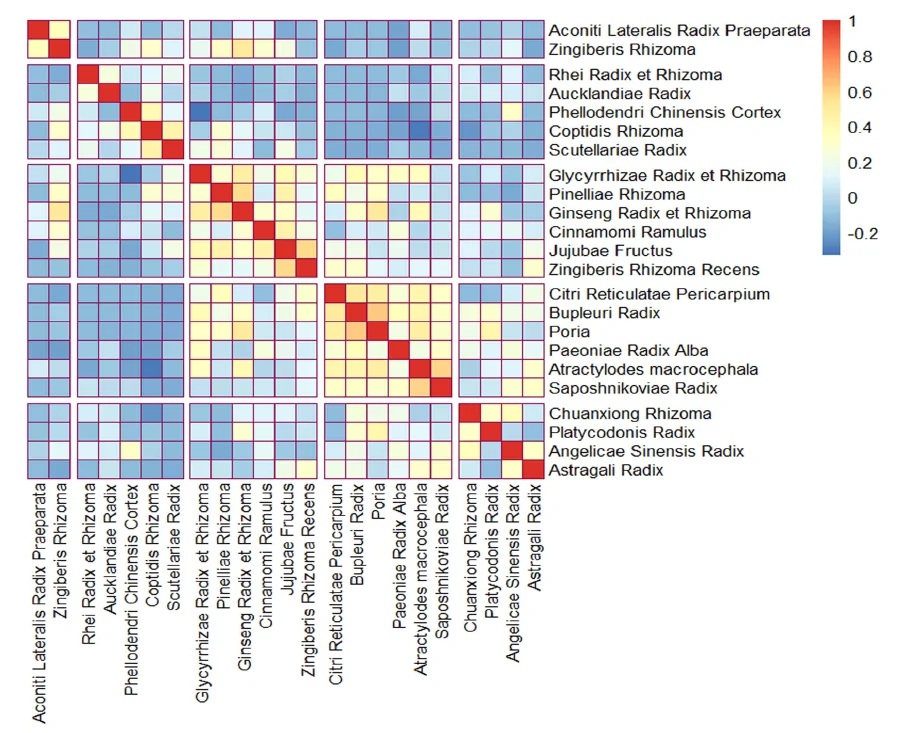
Correlation analysis of high-frequency Chinese medicines.

R Studio was used for phi analysis based on high-frequency TCM with frequency≥5, and the top 20 herb pairs are ranked according to the correlation coefficients, as shown in Table **4**. The top three herb pairs were *Bupleuri- Poria* (0.62), *Atractylodis-Saposhnikoviae* (0.6), *Zingiberis-Jujubae* (0.58). A heat map package was used to indicate a greater degree of similarity between the two herbs in each pair (Fig. **6**).

## 3.6 Mechanism Studies

**Fig. (7):**
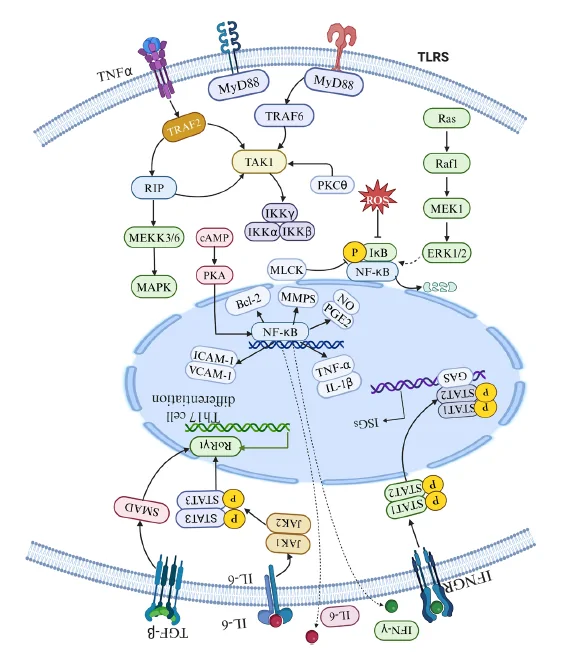
Signal pathway modulated by CP in the treatment of UC.

The analysis revealed that the medicines in CP primarily exert their effects through anti-inflammatory activity, immune modulation, and intestinal barrier repair, reported in 165, 83, and 79 articles, respectively. Key signaling pathways involved in the inflammatory response include the canonical NF-κB pathway, which is activated by proinflammatory factors such as TNF-α, IL-1β, or TLRs, leading to increased production of ICAM, MMPs, PGE_2_, and various cytokines. Intestinal barrier regulation is mainly associated with the NF-κB/MLCK pathway, which modulates the expression of occludin and claudin proteins. Additionally, immune-related targets for UC treatment in CP predominantly involve RORγt and Th17 cell differentiation. RORγt, stimulated by TGF-β or IL-6, drives the expression of RORγt protein and ultimately promotes the formation of Th17 cells with specialized immune functions (Fig. **7** and Table **5**).

## 3.7 Clinical Analysis

The clinical application of several classical prescriptions was analyzed, encompassing a total of 2,045 cases, with individual study sample sizes ranging from 30 to 178. The control group primarily received mesalazine, and the treatment duration varied between 28 and 180 days. Baitouweng decoction, Shenling Baizhu powder, and Sishen pill were mainly used to address the syndromes of damp-heat in the large intestine, spleen deficiency with dampness accumulation, and yang deficiency of the spleen and kidney, respectively. Overall, the clinical efficacy rates of the TCM treatment groups were generally higher than those of the control groups (Table **6**).

## 4 DISCUSSION

In TCM, UC falls under the categories of “chang pi” and “dysentery.” Descriptions related to UC date back to the pre-Qin and Han Dynasties, with “chang pi” recorded in Huangdi's Internal Classic and “dysentery with heat and tenesmus” described in Treatise on Cold Pathogenic and Miscellaneous Diseases, reflecting early understanding of the disease. Principles and prescriptions for UC treatment began appearing in the Eastern Han Dynasty, and after thousands of years of development, TCM has accumulated profound knowledge and treatment advantages for UC. Through systematic analysis of classical prescriptions (CP) for UC, the top three most frequently used were Sishen pill, Huangqin decoction, and Shaoyao decoction. Modern studies have shown that the Sishen pill treats UC by preventing excessive epithelial cell apoptosis, repairing intestinal mucosal damage, regulating intestinal flora, and exerting anti-inflammatory and antioxidant effects [57, 58]. Huangqin decoction, known as the “ancestor prescription for the treatment of dysentery,” contains baicalin and Scutellaria baicalensis Georgi polysaccharides, which play anti-inflammatory roles by inhibiting the expression of key cytokines such as IL-6 [59, 60]. Similarly, Shaoyao decoction has been confirmed to improve mucosal permeability, promote intestinal epithelial cell regeneration, restore intestinal balance, and effectively treat UC [61].

In TCM, the pathology of UC is characterized by deficiency or weakness in the body, invasion by phlegm and blood stasis, and insufficiency of spleen function [62]. During the acute phase, UC is primarily marked by the accumulation of damp heat in the intestines, which is treated mainly by clearing heat and draining dampness. In the remission phase, the condition often presents as a combination of deficiency and excess, with deficiency predominating, and treatment focuses on tonifying the deficiency while clearing heat. Additionally, UC patients with dampness and heat accumulation frequently experience qi stagnation and blood stasis, which aggravate pathological manifestations, and thus herbs that promote blood circulation and remove pathological products are commonly incorporated [63].

Analysis of the frequency of herbs in classical prescriptions for UC revealed that those used more than ten times were predominantly heat-clearing and deficiency-tonifying herbs. During the acute phase, heat-clearing herbs, typically bitter and cold in nature, are frequently prescribed, whereas in the remission phase, deficiency-tonifying herbs, often sweet and warm, are preferred. The sweet flavor corresponds to the spleen meridian and has tonifying and moderating effects, helping regulate the spleen and stomach in the “middle jiao.” It not only provides tonification but also alleviates abdominal pain caused by other drugs. When combined with pungent-flavored herbs, it leverages their ability to promote qi movement, activate blood circulation, and resolve blood stasis, thereby facilitating the elimination of pathological products.

Herb pairs are the fundamental elements of a prescription, and analyzing core herb pairs can provide valuable guidance for clinical prescription selection. In the CP for the treatment of UC, the core herb pairs identified were *Glycyrrhizae Radix et Rhizoma–Ginseng Radix et Rhizoma*, *Atractylodis–Saposhnikoviae*, and *Bupleuri–Poria*.

The combination of *Glycyrrhizae Radix et Rhizoma* and *Ginseng Radix et Rhizoma* strengthens the spleen, transforms dampness, and nourishes the spleen and stomach, showing significant therapeutic effects for UC characterized by spleen deficiency and damp-phlegm obstruction. The *Bupleuri–Poria* pair harmonizes the liver and spleen, which is beneficial for UC patients with spleen deficiency and dampness. *Saposhnikovia dispels* and resolves external pathogens, overcomes dampness, and relieves pain, while *Atractylodes macrocephala dries dampness*, facilitates water flow, strengthens the spleen, and boosts vital energy, thereby supporting overall bodily vitality.

Currently, the control drugs in clinical trials for the treatment of mild to moderate UC are primarily 5-ASA, including mesalazine and sulfasalazine [64]. However, UC patients exhibit varied responses to 5-ASA, and more than 50% show little or no therapeutic effect [65]. In contrast, TCM tailors treatment to different symptom patterns, achieving higher efficacy and response rates in clinical management of UC. Analysis of CP in clinical trials revealed that Baitouweng decoction achieved an efficacy rate of 98.9% in patients with dampness-heat accumulation syndrome. For patients with dampness stagnancy due to spleen deficiency, Shenling Baizhu Powder demonstrated an efficacy rate of 93.33%. Notably, Sishen Pill, used primarily for spleen-kidney yang deficiency syndrome, showed an efficacy rate exceeding 97% and effectively alleviated clinical symptoms in UC patients.

The pathogenesis of UC is not yet fully elucidated, but it is closely associated with inflammatory responses, disruption of the intestinal mucosal barrier, and immune imbalance [66]. Studies have shown that activation of CD4^+^ T cells in UC patients triggers excessive activation of helper T cells and relatively insufficient activation of Treg cells. This leads to a Th1/Th2 imbalance, accelerating UC progression. During intestinal inflammation, proinflammatory cytokines alter the structure of the intestinal epithelium, increasing mucosal permeability [67, 68]. Consequently, luminal bacteria and antigenic substances can migrate into the lamina propria, activating immune cells and exacerbating inflammation [69, 70]. Analysis of the collected literature identified NF-κB, cytokines, MLCK, RORγt, and Th17 cell differentiation as key targets for UC treatment with CP, which play crucial roles in inflammatory response, intestinal barrier integrity, and immune modulation [71, 72]. The NF-κB pathway mediates inflammation by regulating the expression of proinflammatory cytokines, including TNF-α and IL-6 [73], while TGF-β or IL-6 activation of RORγt drives Th17 cell differentiation to modulate immunity [74, 75]. Therefore, targeting and inhibiting these pathways can effectively reduce inflammatory factor release and alleviate intestinal inflammation in UC therapy.

Firstly, these findings are entirely derived from animal experiments, which may not fully capture the complex pathophysiological processes and individual variations seen in human patients. Secondly, the clinical application of these CPs has not been rigorously assessed through a substantial number of randomized controlled trials (RCTs). Without such evidence, it is premature to conclude that these interventions are both effective and safe for human use. Therefore, further research is essential to validate the therapeutic potential of these CPs in the treatment of UC.

## CONCLUSION

In conclusion, data mining revealed that the top three prescriptions for the treatment of UC were the Sishen pill, Huangqin decoction, and Shaoyao decoction, while the most frequently used herbs included *Glycyrrhizae Radix et Rhizoma, Coptidis Rhizoma, Paeoniae Radix Alba,* and *Atractylodis Macrocephalae Rhizoma*. The core herb pairs in CP for UC treatment comprised *Bupleuri–Poria, Atractylodis–Saposhnikoviae, Zingiberis–Jujubae, Phellodendri Chinensis Cortex–Coptidis Rhizoma*, and *Pinelliae Rhizoma–Ginseng Radix et Rhizoma*. Mechanistically, these medicines primarily act by alleviating inflammation, restoring mucosal barriers, and maintaining immune balance. However, it is important to emphasize that these findings are based on animal experiments rather than human studies. Therefore, their efficacy and safety must be validated through randomized controlled clinical trials before being applied in human treatment.

## AUTHORS’ CONTRIBUTIONS

The authors confirm their contribution to the paper as follows: Draft manuscript, Wang J.; data collection: Xu J.H.; analysis and interpretation of results: Zhang M. M.; Study conception and design, Li Y., review & editing: Xiao H. M., Li M. All authors reviewed the results and approved the final version of the manuscript.

## Figures and Tables

**Fig. (1):**
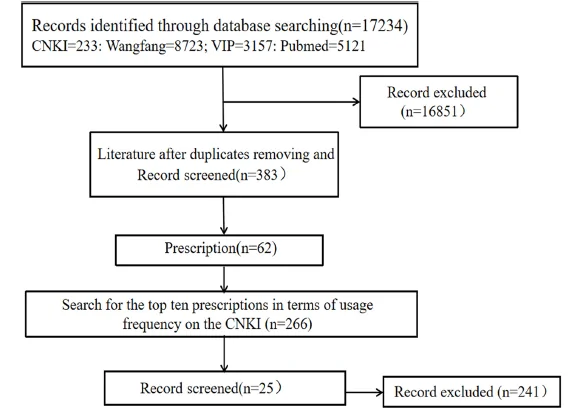
Prisma flow diagram.

**Fig. (4):**
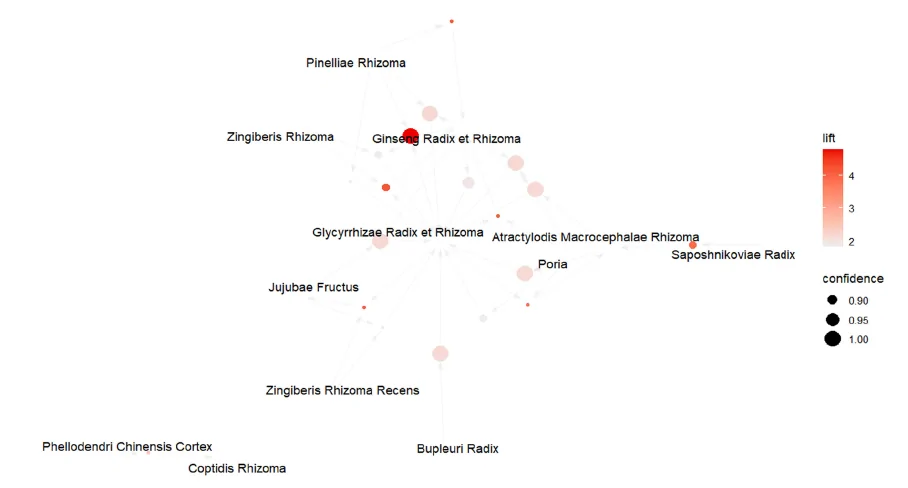
Association rules.

**Table 1:** Related information on classical formulas on the treatment of UC.

Formula	Frequency	Formula Derivation	Duration	Author
Si Shen Pill	39	Zheng Zhi Zhun Sheng	10th year of the Wanli era	Wang Ken Tang
Huang Qin Decoction	37	Shang Han Lun	Eastern Han Dynasty	Zhang Zhong Jing
Shao Yao Decoction	36	Suwen Bingji Qiyi Baoming Ji	Jin Dynasty	Liu Wan Su
Shen Ling Bai Zhu San	32	Tai ping Huimin Heji Ju Fang	Zhenghe era of the Northern Song Dynasty	Song Dynasty Medical Bureau Compilation
Ge Gen Qin Lian Decoction	23	Shang Han Lun	Eastern Han Dynasty	Zhang Zhong Jing
Wu Mei Pill	23	Shang Han Lun	Eastern Han Dynasty	Zhang Zhong Jing
Tongxie Yao Fang	22	Dan Xi Xin Fa	Yuan Dynasty	Zhu Zhenheng
Bai Tou Weng Decoction	20	Shang Han Lun	Eastern Han Dynasty	Zhang Zhong Jing
Ban Xia Xie Xin Decoction	14	Shang Han Lun	Eastern Han Dynasty	Zhang Zhong Jing
Yu Lian Pill	10	Dan Xi Xin Fa	Yuan Dynasty	Zhang Zhong Jing
Si Jun Zi Decoction	8	Taiping Huimin Heji Ju Fang	Zhenghe era of the Northern Song Dynasty	Song Dynasty Medical Bureau Compilation
Yi Yi Fu Zi Bai Jiang San	7	Jin Gui Yao Lue	3rd century AD	Zhu Zhen Heng
Gu Chang Zhi Xie	7	2020 edition pharmacopeia	/	/
Xiang Lian Pill	7	Military Department's Collection of Formulas	Tang Dynasty	Li Jiang
Gan Cao Xie Xin Decoction	6	Shang Han Lun	Eastern Han Dynasty	Zhang Zhong Jing
Ren Shen Bai Du San	6	Tai Ping Huimin Heji Ju Fang	Zhenghe era of the Northern Song Dynasty	Song Dynasty Medical Bureau Compilation
Da Huang Mu Dan Decoction	5	Jin Gui Yao Lue	3rd century AD	Zhang Zhong Jing
Huai Hua San	4	Pu Ji Ben Shi Fang	Song Dynasty	Xu Shu Wei
Bu Zhong Yi Qi Pill	4	Nei Wai Shang Bian Huo Lun	Yuan Dynasty	Li Dong Yuan
Bu Pi Yi Chang Pill	4	2020 edition pharmacopeia	/	/
Si Ni San	4	Shang Han Lun	Eastern Han Dynasty	Zhang Zhong Jing
Xiang Shen Pill	4	Qi Fang Lei Bian	Qing Dynasty	Wu Shi Chang
Huang Lian Jie Du Decoction	4	Zhu Hou Bei Ji Fang	Eastern Jin Dynasty	Ge Hong
Li Zhong Decoction	4	Shang Han Lun	Eastern Han Dynasty	Zhang Zhong Jing
Cang Fang Decoction	3	Suwen Bingji Qiyi Baoming Ji	Jin Dynasty	Liu Pill Su
Wei Chang An Pill	3	2020 edition pharmacopeia	/	/
Si Ni Decoction	3	Shang Han Lun	Eastern Han Dynasty	Zhang Zhong Jing
Ping Wei San	3	2020 edition pharmacopeia	/	/
Xi Lei San	3	Jin Gui Yi	Qing Dynasty	You Zaijing
Chai Shao Liu Jun Zi	2	Yi Zong Jin Jian	Qing Dynasty	Wu Qian
Dang Gui Bu Xue Decoction	2	Nei Wai Shang Bian Huo Lun	Southern Song Dynasty	Li Dongyuan
Xiao Jian Zhong Decoction	2	Shang Han Lun	Eastern Han Dynasty	Zhang Zhong Jing
San Huang Xie Xin Decoction	2	Jin Gui Yao Lue	3rd century AD	Zhang Zhong Jing
Huang Lian Hou Po	2	Pu Ji Ben Shi Fang	Song Dynasty	Xu Shuwei
San Ao Decoction	1	Jin Gui Yao Lue	3rd century AD	Zhang Zhong Jing
Shu Yu Pill	1	Jin Gui Yao Lue	3rd century AD	Zhang Zhong Jing
Xie Li Decoction	1	Yi Xue Zhong Zhong Can Xi Lu	Qing Dynasty	Zhang Xi Chun
Qu Shi Jian Pi Decoction	1	Pill Bing Hui Chun	Ming Dynasty	Gong Ting Xian
Bao He Pill	1	2020 edition pharmacopeia	/	/
Tao Hong Si Wu Decoction	1	Yi Zong Jin Jian	Qing Dynasty	Wu Qian
Sheng Yang Yi Wei Decoction	1	Nei Wai Shang Bian Huo Lun	Southern Song Dynasty	Li Dongyuan
San Huang Shou Ai Decoction	1	Book of Living Proofs for Diverse Diseases	Song Dynasty	Zhu Gong
Si Wu Decoction	1	Xian Shou Li Shang Xu Duan Mi Fang	Tang Dynasty	Lin Daoren
Xin Huang Pian	1	2020 edition pharmacopeia	/	/
Er Shen Pill	1	Pu Ji Ben Shi Fang	Song Dynasty	Xu Shuwei
Fu Zi Gan Jiang	1	Pu Ji Fang	Ming Dynasty	Zhu Fu
Zhi Kang Capsule	1	2020 edition pharmacopeia	/	/
Sheng Jiang Xie Xin Decoction	1	Shang Han Lun	Eastern Han Dynasty	Zhang Zhong Jing
Huang Qi Jian Zhong Pill	1	Jin Gui Yao Lue	3rd century AD	Zhang Zhong Jing
Zhi Tong Ru Shen Decoction	1	Wai Ke Qi Xuan	Ming Dynasty	Shen Douyuan
Shao Yao Gan Cao Decoction	1	Shang Han Lun	Eastern Han Dynasty	Zhang Zhong Jing
Fu Zi Li Zhong Pill	1	Tai Ping Huimin Heji Ju Fang	Zhenghe era of the Northern Song Dynasty	Song Dynasty Medical Bureau Compilation
Huang Lian Gan Jiang Decoction	1	Dan Xi Xin Fa	Yuan Dynasty	Zhu Zhenheng
Gan Jiang Decoction	1	Bei Ji Qian Jin Yao Fang	Tang Dynasty	Sun Simiao
San Qi Xue Shang Ning	1	2020 edition pharmacopeia	/	/
Tou Nong San	1	Wai Ke Zheng Zong	Ming Dynasty	Chen Shigong
Xian He Cao Chang Yan	1	2020 edition pharmacopeia	/	/
Pai Nong San	1	Jin Gui Yao Lue	3rd century AD	Zhang Zhong Jing
Huang Tu Decoction	1	Jin Gui Yao Lue	3rd century AD	Zhang Zhong Jing
Chang Yan Ning	1	2020 edition pharmacopeia	/	/
Yu Ping Feng San	1	Dan Xi Xin Fa	Yuan Dynasty	Zhu Zhenheng
Huang Lian Decoction	1	Shang Han Lun	Eastern Han Dynasty	Zhang Zhong Jing

**Table 2:** Herbs’ Chinese, Latin name frequency and probability.

**No.**	**Chinese**	**Latin name**	**Frequency (times)**	**Probability (%)**
1	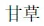	*Glycyrrhizae Radix et Rhizoma*	29	46.77%
2	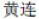	*Coptidis Rhizoma*	21	33.87%
3	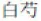	*Paeoniae Radix Alba*	16	25.81%
4	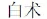	*Atractylodis Macrocephalae Rhizoma*	14	22.58%
5	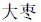	*Jujubae Fructus*	13	20.97%
6	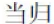	*Angelicae Sinensis Radix*	13	20.97%
7	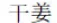	*Zingiberis Rhizoma*	13	20.97%
8	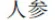	*Ginseng Radix et Rhizoma*	13	20.97%
9	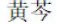	*Scutellariae Radix*	10	16.13%
10	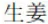	*Zingiberis Rhizoma Recens*	9	14.52%
11	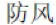	*Saposhnikoviae Radix*	8	12.90%
12	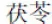	*Poria*	8	12.90%
13	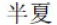	*Pinelliae Rhizoma*	7	11.29%
14	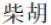	*Bupleuri Radix*	7	11.29%
15	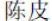	*Citri Reticulatae Pericarpium*	7	11.29%
16	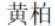	*Phellodendri Chinensis Cortex*	7	11.29%
17	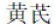	*Astragali Radix*	7	11.29%
18	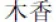	*Aucklandiae Radix*	7	11.29%
19	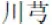	*Chuanxiong Rhizoma*	6	9.68%
20	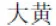	*Rhei Radix et Rhizoma*	6	9.68%
21	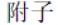	*Aconiti Lateralis Radix Praeparata*	6	9.68%
22	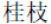	*Cinnamomi Ramulus*	5	8.06%
23	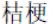	*Platycodonis Radix*	5	8.06%

**Table 3:** Association rules of herbs for UC treatment.

**No.**	**LHS (Left Hand Side)**	**RHS (Right Hand Side)**	**Support**	**Confidence**	**Lift**
1	*{Glycyrrhizae Radix et Rhizoma, Pinelliae Rhizoma}*	*{Ginseng Radix et Rhizoma }*	0.097	1.000	4.769
2	*{Glycyrrhizae Radix et Rhizoma, Zingiberis Rhizoma}*	*{Ginseng Radix et Rhizoma }*	0.113	0.875	4.173
3	*{Pinelliae Rhizoma}*	*{Ginseng Radix et Rhizoma }*	0.097	0.857	4.088
4	*{Glycyrrhizae Radix et Rhizoma, Zingiberis Rhizoma Recens}*	*{Jujubae Fructus}*	0.097	0.857	4.088
5	*{Glycyrrhizae Radix et Rhizoma, Poria}*	*{Ginseng Radix et Rhizoma }*	0.097	0.857	4.088
6	*{Saposhnikoviae Radix}*	*{Atractylodis Macrocephalae Rhizoma}*	0.113	0.875	3.875
7	*{Glycyrrhizae Radix et Rhizoma, Poria}*	*{Atractylodis Macrocephalae Rhizoma}*	0.097	0.857	3.796
8	*{Phellodendri Chinensis Cortex}*	*{Coptidis Rhizoma}*	0.097	0.857	2.531
9	*{Bupleuri Radix}*	*{Glycyrrhizae Radix et Rhizoma}*	0.113	1.000	2.138
10	*{Ginseng Radix et Rhizoma, Pinelliae Rhizoma}*	*{Glycyrrhizae Radix et Rhizoma}*	0.097	1.000	2.138

**Table 4:** The top 20 herb pairs of high-frequency TCM phi.

**No.**	**Herb Combination**	**phi**	**No.**	**Herb Combination**	**phi**
1	*Bupleuri-Poria*	0.62	11	*Pinelliae-Jujubae*	0.44
2	*Atractylodis-Saposhnikoviae*	0.6	12	*Cinnamomi-Jujubae*	0.43
3	*Zingiberis-Jujubae*	0.58	13	*Coptidis-Scutellariae*	0.43
4	*Pinelliae-Ginseng*	0.57	14	*Atractylodis-Bupleuri*	0.42
5	*Bupleuri-Citri*	0.52	15	*Atractylodis-Citri*	0.42
6	*Ginseng-Poria*	0.51	16	*Poria-Platycodonis*	0.42
7	*Ginseng-Zingiberis*	0.51	17	*Atractylodis-Ginseng*	0.39
8	*Atractylodis-Poria*	0.48	18	*Glycyrrhizae-Jujubae*	0.39
9	*Citri-Poria*	0.47	19	*Coptidis-Phellodendri*	0.39
10	*Glycyrrhizae-Ginseng*	0.47	20	*Bupleuri-Glycyrrhizae*	0.38

**Table 5:** Analysis of related signaling pathways of traditional chinese medicine in the treatment of ulcerative colitis.

Primary Mechanism	Number of Publications	Main Targets	Related Signaling Pathways	Main Reference
Anti-inflammatory effects	165	TLR4,PPARγ, IL-1β,TNF-α IL-6,MyD88,NF-κB,MMP-7	TLR4/MyD88/NF-κB TLR4/PPARγ/NF-κB/NLRP3	[23] [24] [25]
Modulation of immunity	83	STAT3,IL-6,TGF-β,RORγt,JAK,Foxp3	IL-6/JAK2/STAT3 Th17/Treg Cell Homeostasis Regulation	[26] [27] [28]
Repairing the intestinal barrier	79	ZO-1,Occludin,Claudin-1,MLCK,p-MLC,ILC-3	ILC3/IL-22/IL-17A miR-185-3p MLCKp-MLC TLR4/NF-κB/MLCK	[29] [30] [31]

**Table 6:** Clinical trial research on classical famous prescriptions for treating UC.

**Medicine**	**Case Number**	**Syndrome Type**	**Medication**		**Total Effective Rate (%)**		**Treatment Course(days)**	**Reference**
			**Treatment Group**	**Control Group**	**Treatment Group**	**Control Group**		
Baitouweng Decoction	62	Syndrome of dampness-heat in the large intestine	Baitouweng Decoction	Mesalazine	93.80%	96.7%	56	[32]
	110			Mesalazine	90.91%	74.55%	28	[33]
	66			Mesalazine	88.60%	64.50%	28	[34]
	60			Prednisone + Sulfasalazine	96.7%	76.70%	28	[35]
	47			Mesalazinek	93.62%	76.60%	56	[36]
	68		Modified Baitouweng Decoction	Sulfasalazine	97.10%	76.50%	60	[37]
	94			Mesalazine	91.49%	63.83%	28	[38]
	34			Sulfasalazine	94.11%	76.47%	45	[39]
	156			Mesalazine	96.20%	84.60%	60	[40]
	70			Mesalazine + Kangfuxin Liquid	94.28%	77.14%	20	[41]
	86			Mesalazine + Placebo	95.35%	74.42%	28	[42]
	168			Olsalazine Sodium Capsules	98.9%	71.80%	30	[43]
	80			Mesalazine	95%	70.00%	56	[44]
	102			Mesalazine	90.38%	74.00%	56	[45]
	120			Kangfuxin Liquid	93.30%	63.30%	28	[46]
Shenling Baizhu Powder	178	Syndrome of spleen deficiency and dampness accumulation	Shenling Baizhu Powder	/	94.38%	/	90-180	[47]
	92			Peifeikang	93.48%	76.09%	28	[48]
	30			/	96.67%	/	40-80	[49]
	67			levofloxacin injection + Metronidazole Injection	97%	81.00%	42	[50]
	48			Mesalazine	91.67%	70.83%	90	[51]
	60			Mesalazine	93.33%	66.67%	84	[52]
Sishen Pill	45	Syndrome of yang deficiency of the spleen and kidney	Sishen Pill	/	97.80%	/	/	[53]
	30			Sulfasalazine + Prednisone	93.33%	73.33%	30	[54]
	86			Clostridium butyricum Tablets	95.30%	72.10%	28	[55]
	86			Sulfasalazine	100%	97.73%	30	[56]

## LIST OF ABBREVIATIONS

UC: Ulcerative Colitis
CP: Classic Prescription
TCM: Traditional Chinese Medicine
